# Optimization of the dye-sensitized solar cell performance by mechanical compression

**DOI:** 10.1186/1556-276X-9-523

**Published:** 2014-09-23

**Authors:** Teen Hang Meen, Jenn Kai Tsai, Yu Shin Tu, Tian Chiuan Wu, Wen Dung Hsu, Shoou-Jinn Chang

**Affiliations:** 1 Department of Electronic Engineering, National Formosa University, Yunlin 632, Taiwan; 2 Department of Materials Science and Engineering, National Cheng Kung University, Tainan 701, Taiwan; 3 Institute of Microelectronics and Department of Electrical Engineering, Center for Micro/Nano Science and Technology, Advanced Optoelectronic Technology Center, National Cheng Kung University, Tainan 701, Taiwan

**Keywords:** Mechanical compression, Dye-sensitized solar cells (DSSCs), TiO_2_, Doctor blade method, Conversion efficiency

## Abstract

In this study, the P25 titanium dioxide (TiO_2_) nanoparticle (NP) thin film was coated on the fluorine-doped tin oxide (FTO) glass substrate by a doctor blade method. The film then compressed mechanically to be the photoanode of dye-sensitized solar cells (DSSCs). Various compression pressures on TiO_2_ NP film were tested to optimize the performance of DSSCs. The mechanical compression reduces TiO_2_ inter-particle distance improving the electron transport efficiency. The UV–vis spectrophotometer and electrochemical impedance spectroscopy (EIS) were employed to quantify the light-harvesting efficiency and the charge transport impedance at various interfaces in DSSC, respectively. The incident photon-to-current conversion efficiency was also monitored. The results show that when the DSSC fabricated by the TiO_2_ NP thin film compressed at pressure of 279 kg/cm^2^, the minimum resistance of 9.38 Ω at dye/TiO_2_ NP/electrolyte interfaces, the maximum short-circuit photocurrent density of 15.11 mA/cm^2^, and the photoelectric conversion efficiency of 5.94% were observed. Compared to the DSSC fabricated by the non-compression of TiO_2_ NP thin film, the overall conversion efficiency is improved over 19.5%. The study proves that under suitable compression pressure the performance of DSSC can be optimized.

## Background

In recent years, most of the solar cells are fabricated on Si-based substrate [1] which is flat and rigid; however, several good substitutes have been discovered to replace Si-based substrates for applications in flexible solar panels. Dye-sensitized solar cell (DSSC) is one of alternatives as it can be built on flexible substrate with low cost and light weight [2-4]. Grätzel and his colleagues published the first DSSC in 1992 [2]. They adopted nanoporous titanium dioxide (TiO_2_) as photoanode, metal ruthenium (Ru) organic complexes as dyes, and I_2_/I_3_^−^ redox couple as electrolyte. The photoelectric conversion efficiency for the first DSSC is 7.1%. Since then, improving the performance of dye-sensitized solar cell has been the primary goal of researchers.

Dye-sensitized solar cell has three main components: (i) the dye which absorbs solar energy to generate excitons [5,6], (ii) the nanostructured metal oxide (photoanode) which captures electrons from the dye [7,8], and (iii) the redox electrolyte which transports electrons and holes from metal oxide and oxidized dye to electrodes [9,10]. Therefore, the conversion efficiency of dye-sensitized solar cell is mainly influenced by transparent conductive oxide (TCO) (photoanode), sensitizer (dye), electrolyte, electrodes, etc.

The photoanode is usually made by TiO_2_ nanoparticles due to its chemical stability, non-toxicity, relatively high transmittance in the visible spectral, etc. It has been proven a good photoelectric material. The band gaps of rutile- and anatase-phased TiO_2_ are 3.0 and 3.2 eV, respectively. The anatase phase is more preferred to be used in the DSSC owing to its photocatalytic property and wide direct band gap [11].

Literatures have shown that the thickness of TiO_2_ nanoparticle (NP) thin film plays an important role on the conversion efficiency of DSSC. However, using nanoparticles as a carrier for dye molecules to absorb light hinders the electron transport to anode electrode, due to the loss of electrons by either recombination between electrons and the oxidized dye molecules or electron-accepting species in the electrolyte or back reaction during the transport processes [12-14].

**Figure 1:**
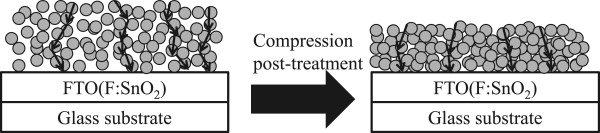
**Deposition of TiO**_**2 **_**films by doctor blade process and a subsequent mechanical compression as post-treatment.**

Mechanical compression of TiO_2_ NP thin film to reduce the film thickness could be an effective method to suppress the recombination without changing the mass and effective surface area of TiO_2_ NPs. The reduction of the thickness helps reducing the electron transport path and internal impedance [15,16]. Schematic diagram of compression effect is shown in Figure 1.

Previously, we have reported that the performance of TiO_2_ NP thin films can be improved by mechanical compression, but the compression pressure was not optimized [12]. In this study, the effect of compression pressure on the TiO_2_ NP thin films was studied in detail. The structures and morphologies of TiO_2_ NP thin films were characterized using field-emission scanning electron microscope (FE-SEM). The UV–vis spectrophotometer and electrochemical impedance spectroscopy (EIS) were employed to quantify the light-harvesting efficiency and the charge transport impedance at various interfaces in DSSC, respectively. The incident photon-to-current conversion efficiency was also monitored. The I-V characterization was performed under standard air mass 1.5 global (AM 1.5 G) simulated sunlight.

## Methods

### Experimental details

#### Deposition of TiO_2_ thin films as photoanode

TiO_2_ paste of 10 wt% was prepared by mixing nanocrystalline TiO_2_ particles (TG-P25, Degussa, Shinjuku, Tokyo, Japan; the average nanoparticle diameter was about 25 to 30 nm) with *tert*-butyl alcohol and deionized water. TiO_2_ paste was scraped on a transparent fluorine-doped-tin oxide (FTO) glass with resistivity of 8 Ω/sq by the doctor blade method to form TiO_2_ NP thin film. Subsequently, the mechanical compression was performed on the film. The TiO_2_ NP thin film then was annealed in two steps in air: under 150°C for 90 min and then under 500°C for 30 min. The 150°C annealing was set for decomposition of residual organic compounds, and the 500°C annealing was used to assist the interconnection of TiO_2_ NPs, so that the loss of electrons during transport becomes less. In this study, various pressures, 61, 131, 279, 558, and 858 kg/cm^2^, were adopted to compress the TiO_2_ NP thin film, named samples B to F, respectively. The as-deposited film, sample A, was used as a control group.

#### DSSC fabrication

**Figure 2:**
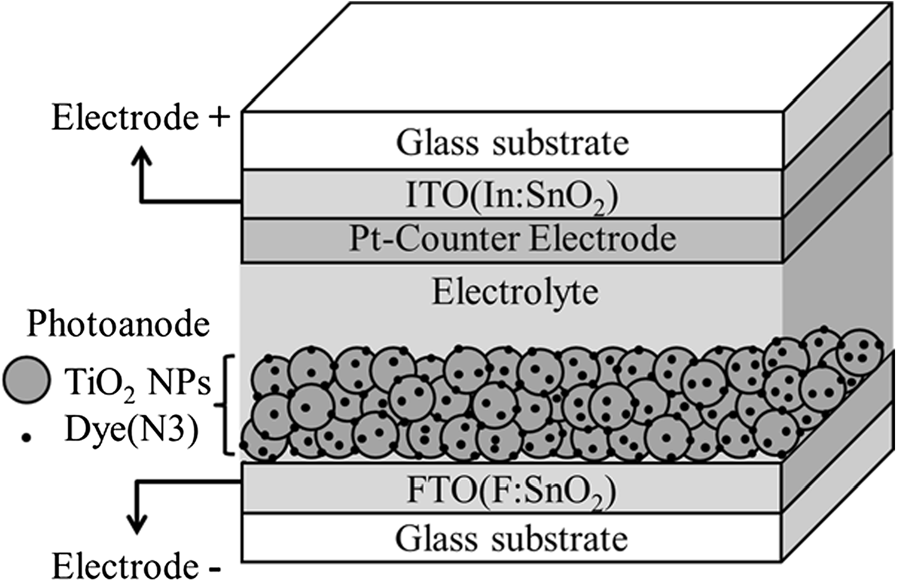
**The schematic diagram of dye-sensitized solar cell with/without compressed TiO**_**2 **_**nanoparticle thin film as photoanode.**

A schematic diagram of dye-sensitized solar cell with/without compressed TiO_2_ NP thin film as photoanode is shown in Figure 2. The FTO substrate coated with TiO_2_ NP thin film were immersed in 0.3 mM N3 dye (*cis*-bis(dithiocyanato)-bis(4,4′-dicarboxylic acid-2,2′-bipyridine) ruthenium (II)) solution for 2 h to fabricate working electrode. Subsequently, the electrode was rinsed in acetonitrile for a few seconds to wash out unbound dyes and dried in the oven at 45°C. The counter electrode was fabricated by a grown 1-nm thick Pt thin film on indium tin oxide (ITO) glass with electroplating method. The working electrode then bonded to a counter electrode by a 50-μm thick hot-melt polymer spacer. Sealing was accomplished by pressing the two electrodes together at 115°C for a few seconds. The redox electrolyte, consisting of 0.5 M LiI, 0.05 M I_2_, and 0.5 M 4-*tert*-butylpyridine (TBP), and 1 M 1-propy1-2,3-dimethylimidazolium (DMPII) mixed with 3-methoxypropionitrile (MPN), was injected into the cell by capillary force through an injecting hole previously mechanically drilled on the counter electrode. Finally, the hole was covered and sealed with a piece of hot-melt polymer to prevent the leakage of fluid-type electrolyte. The resulting active electrode area was approximately 0.25 cm^2^ (0.5 × 0.5 cm).

#### Characterizations and photoelectrochemical measurement

The structures and morphologies of TiO_2_ NP thin films were measured using the field-emission scanning electron microscope (FE-SEM; JSM-7500 F, JEOL, Akishima-shi, Japan). Porosity of TiO_2_ NPs was analyzed by ImageJ program from FE-SEM images [17]. The program is a public domain Java image processing program from the National Institute of Mental Health (NIMH), Bethesda, MD, USA. The ultraviolet–visible (UV–vis) transmittance spectrum was observed by a UV–vis spectrophotometer (U-2900, Hitachi High-Technologies Corporation, Tokyo, Japan) with the wavelength from 300 to 800 nm at room temperature. Electrochemical impedance spectroscopy (EIS; Zahner Zennium, Kronach, Germany) measures the current response while applying an AC voltage as a function of frequency. The frequency ranged from 10 mHz to 100 kHz under illumination of AM 1.5 G at biased open-circuit voltage. The incident photon-to-current conversion efficiency (IPCE) that used to determine the light-harvesting efficiency of the dye, the quantum yield of electron injection, and the efficiency of collecting the injected electrons was recorded using an IPCE instrument equipped with a 1000-W Xenon arc lamp as the light source that composed of a compact 1/8 meter monochromator (CM110, Spectral Products, Putnam, CT, USA), a color filter wheel (CFW-1-8, Finger Lakes Instrumentation, Lima, NY, USA), and a calibrated photodiode (FDS1010-CAL, Thorlabs Inc., Newton, NJ, USA). The IPCE data were taken by illuminating monochromatic light on the solar cells (with the wavelength from 300 to 800 nm) and performed with a source meter (2400, Keithley Instruments, Inc., Cleveland, OH, USA). The current–voltage characteristics were measured by Keithley 2400 (Keithley Instruments, Inc., Cleveland, OH, USA) source meter under a simulated sunlight (SAN-EI XES-40S1, San Ei Brand, Higashi-Yodogawa, Japan), AM 1.5 G radiation at 100 mW/cm^2^.

## Results and discussion

**Figure 3:**
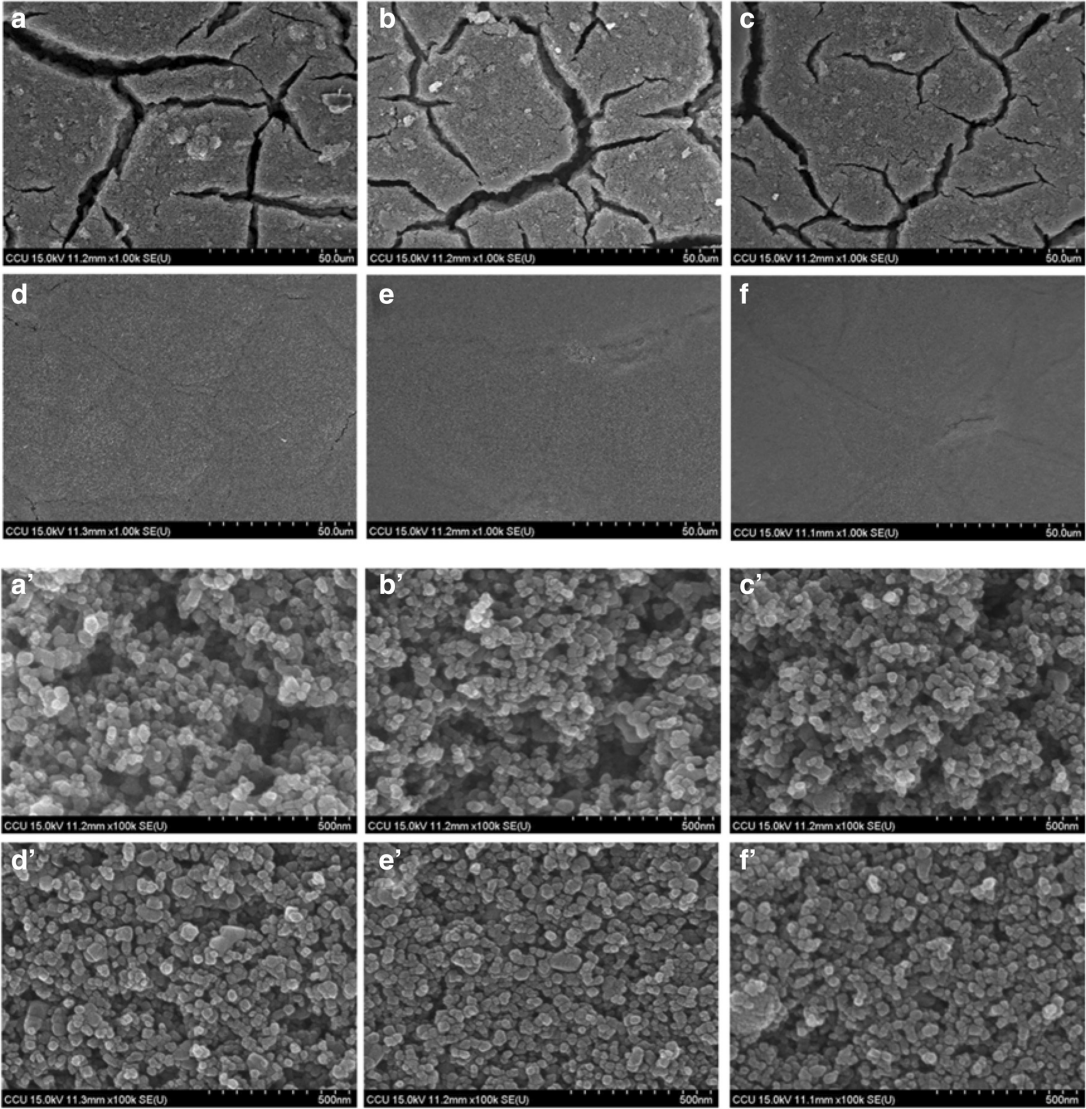
**FE-SEM micrographs for TiO**_**2 **_**nanoparticles film on FTO glass fabricated by doctor blading method with/without compression treatment. (a)** As-deposited film and **(b-f)** after mechanical compression at various pressures, 61, 131, 279, 558, and 858 kg/cm^2^, respectively. The panels **(a’-f’)** show the high magnification images.

Figure 3 shows the FE-SEM image of TiO_2_ NP thin films with and without mechanical compression. Figure 3a shows the surface morphology of the as-deposited TiO_2_ NP (sample A) thin film. There are large cracks observed. Several mechanisms have been proposed to explain the crack formation, such as capillary force that appears when solvent rapidly evaporate from film surface during drying process, decreasing of bond strength among TiO_2_ NPs when the film is thick, and large mismatch of thermal expansion coefficient between the FTO substrate and TiO_2_ NP thin film [18,19]. Figure 3b,c,d,e,f shows the surface morphology of TiO_2_ NP thin film after mechanical compression at 61, 131, 279, 558, and 838 kg/cm^2^ (samples B-F), respectively. As the compression pressure increases, the surface becomes smooth. The surface crack is almost not observed while the compression pressure is 279 kg/cm^2^, as shown in Figure 3d. The film become completely uniform and crack-free when the compression pressure is above 558 kg/cm^2^, as shown in Figure 3e,f. Figure 3a’,b’,c’,d’,f’, which shows the magnification images, demonstrates that the distance between TiO_2_ NPs is gradually decreasing when the compression pressure increases. There, however, are still enough spaces between TiO_2_ nanoparticles for the dye molecules to adhere and electrolyte to permeate.

**Figure 4:**
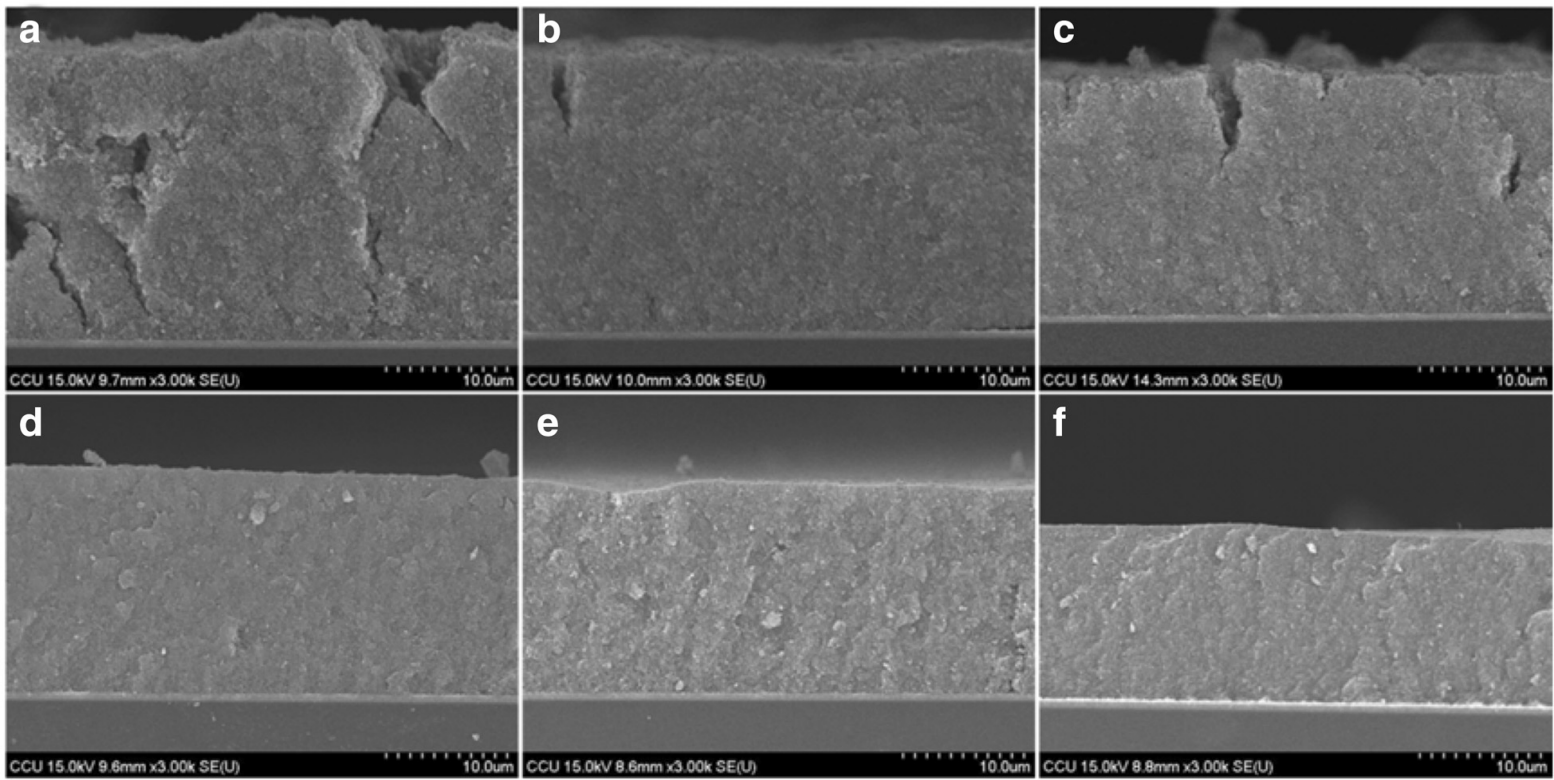
**Cross-sectional images of FE-SEM of TiO**_**2 **_**nanoparticles film with and without compression treatment. (a)** As-deposited film and **(b-f)** after mechanical compression at various pressures, 61, 131, 279, 558, and 858 kg/cm^2^ with the thickness of 24.0, 23.6, 20.2, 18.4, 17.2, and 14.4 μm, respectively.

Figure 4 shows the cross-section images of TiO_2_ NP thin film after compression with different pressures. Figure 4a shows the crack of as-deposited TiO_2_ NP thin film (sample A) that penetrates from the film surface to the FTO substrate. The cracks are gradually mitigated when the compression pressure increases as shown in Figure 4b,c,d,f. When the compression pressure reaches 279 kg/cm^2^ (Figure 4d), there are no obvious cracks observed. The results indicate that post compression is an effective method to synthesis crack-free TiO_2_ NP thin film. The thickness of the TiO_2_ NP thin film also reduces as the compression pressure increases. The thicknesses are 24.0, 23.6, 20.2, 18.4, 17.2, and 14.4 μm when the compression pressures are 0, 61, 131, 279, 558, and 858 kg/cm^2^, respectively. Therefore, the distance between TiO_2_ nanoparticles is short for condensed films. It is then expected that the transport path of the electrons also becomes short, which making them easier to research anode electrode [18].

**Figure 5:**
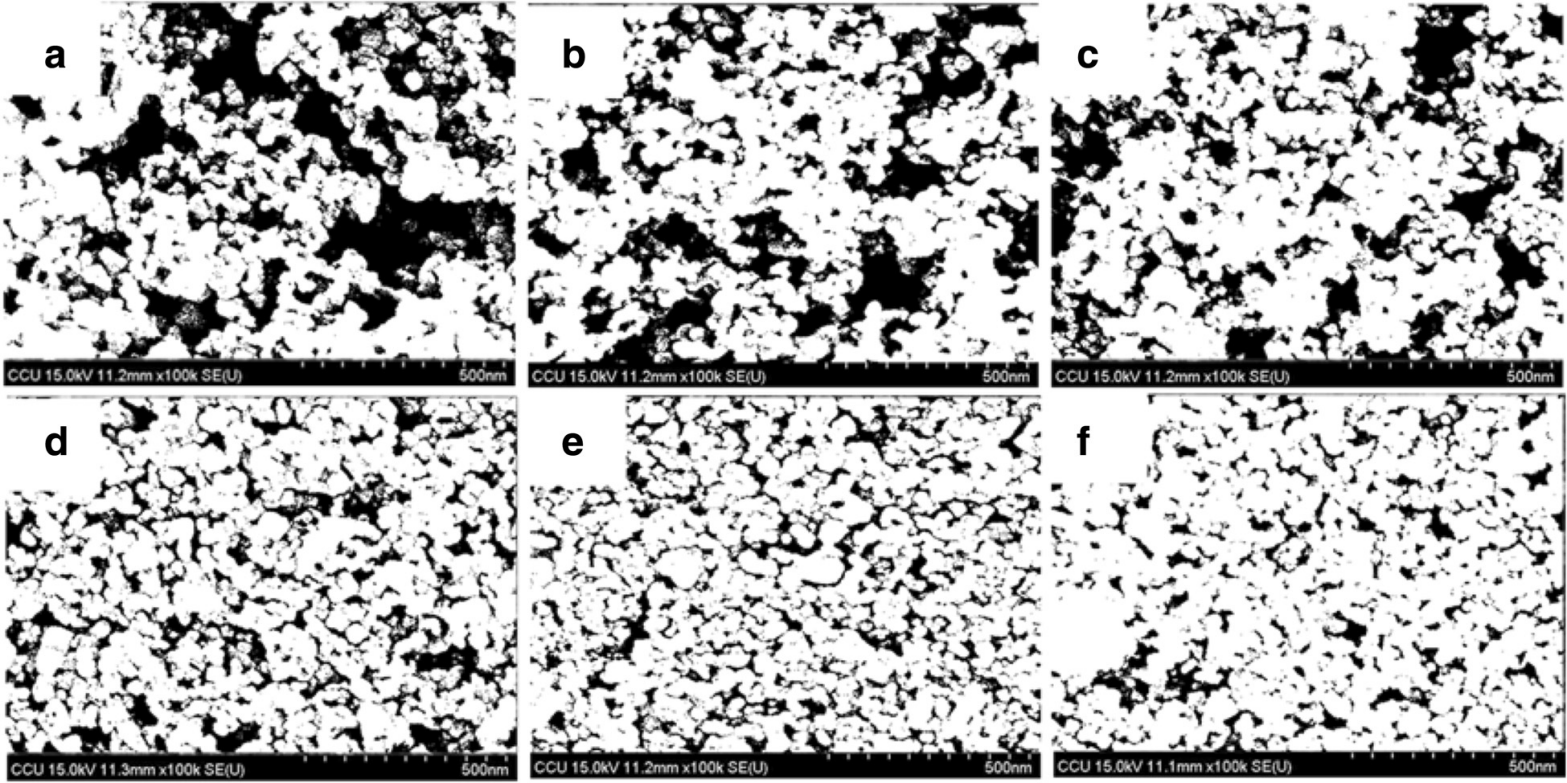
**FE-SEM images analyzed by the ImageJ program.** The black area means the unfilled space, and the white area means the TiO_2_ NPs. **(a)** As-deposited film and **(b-f)** after mechanical compression at various pressures, 61, 131, 279, 558, and 858 kg/cm^2^.

**Figure 6:**
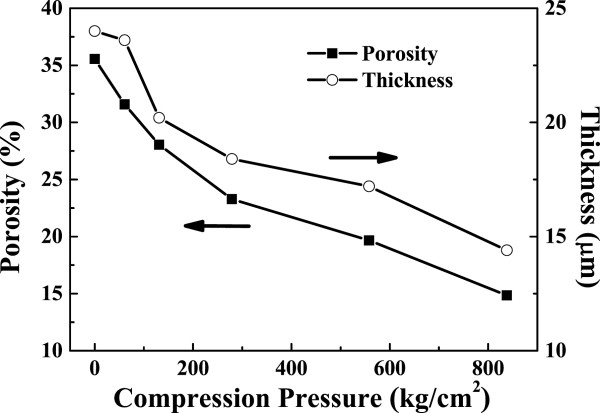
**Thickness and porosity of TiO**_**2 **_**NP thin films as a function of the compression pressure.**

Figure 5 shows the FE-SEM images from Figure 3(a’,b’,c’,d’,e’,f’) after being analyzed by the ImageJ program in order to quantify the porosity of TiO_2_ NP thin film. The black area represents the unfilled space, and the white area represents the TiO_2_ NPs. The images show that the black area decreases as the compression pressure increases. Figure 6 shows both the thickness and porosity of TiO_2_ NP thin film as a function of the compression pressure. The thickness and porosity of TiO_2_ NP thin films decrease from 24.0 to 14.4 μm and 35.56 to 14.85%, respectively, as the compression pressure increases from 0 to 858 kg/cm^2^. The result indicates that the compression treatment not only reduces the thickness but also decreases the porosity to have small inter-particle distance.

**Figure 7:**
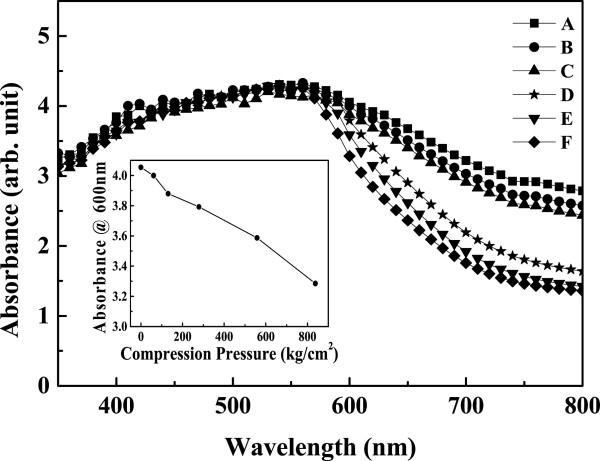
**UV–vis absorption spectra of TiO**_**2 **_**nanoparticle thin film with and without compression treatment.** Curve A is the as-deposited film, and curves B to F are the after mechanical compression at various pressures, 61, 131, 279, 558, and 858 kg/cm^2^.

Figure 7 shows the UV–vis absorption spectra of samples A to F with dye absorption. The absorbance is offset to that of air so that the absorbance of air was set to be zero. At the wavelength shorter than 560 nm, the absorbances are almost the same for all the samples, however, at the wavelength longer than 560 nm, the absorbance decreases as the post-compression pressure increases. Inset of Figure 7 shows the decrease of absorbance at wavelength of 600 nm as the compression pressure increases. The absorbance is due to light absorption by the N3 dye molecules that adhere on the TiO_2_ NP surface. The same absorbance of all the samples at the wavelength less than 560 nm indicates that the number of dye molecules on the TiO_2_ NP surface is almost the same for all the samples even though they have been treated with different compression pressures. Hence, the mechanical compression treatment reduces the inter-particle spaces, and the dye molecules are still allowed to permeate through the TiO_2_ NPs and adhere on their surface. The decrease of absorbance for high-pressure compressed samples at the wavelength longer than 560 nm is attributed to high light-transmission rate for TiO_2_ NP thin film. The samples without being compressed with high pressure also show cracks (Figures 3a,b,c and 4a,b,c), which enhance light scattering resulting increase of light traveling distance in the film and promoting light absorbance.

**Figure 8:**
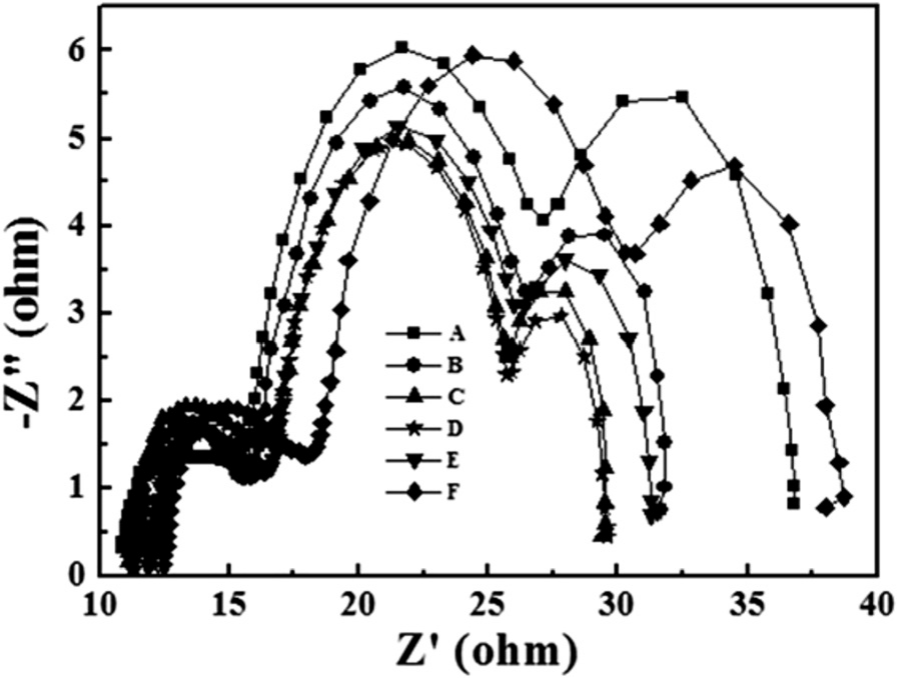
**Nyquist plots of DSSCs made by TiO**_**2 **_**nanoparticle thin film with and without post-compression treatment.** Curve A is the as-deposited film, and curves B to F are the after mechanical compression at various pressures, 61, 131, 279, 558, and 858 kg/cm^2^.

Figure 8 shows the electrochemical impedance spectroscopy (Nyquist plot) of samples A to F after fully fabricated as dye-sensitized solar cells. The Nyquist plot demonstrates the minus imaginary part of impedance (−Z^″^) as a function of the real part of the impedance (Z^′^), while the measured frequency swept from 10 mHz to 100 kHz. The method is very useful to understand the role of interfaces during electron transport. Three distinct semicircles are observed, which are attributed to the electrochemical reaction at the Pt counter electrode/electrolyte interface, charge transport through the dye/TiO_2_ NP/electrolyte interfaces, and the Warburg diffusion process of I^−^/I_3_^−^ in the electrolyte from left to right. The values of the electrochemical impedance for samples A to F are listed in Table 1. The diameter of the first (left-most) semicircle refers to the resistance (*R*_Pt_) at the Pt counter electrode/electrolyte interface. The values are almost the same for all the samples indicating that the fabrication conditions are consistent. The diameter of the second (middle) semicircle corresponds to the resistance (*R*_K_) associated with transport of electrons through dye/TiO_2_ NP/electrolyte interfaces. The value is minimum for sample D, indicating that the recombination probability between electrons and the iodides of the electrolyte is lowest in this case [20]. The diameter of the third (right-most) semicircle (*R*_D_) represents the impedance of the I_3_^−^ ion diffusion in the electrolyte. Since the diffusion of I_3_^−^ ions is suppressed as the electrolyte is confined between two very close TiO_2_ nanoparticles, the condensed TiO_2_ NP thin film would have large *R*_D_. On the other hand, thick TiO_2_ NP thin film would increase the distance for I_3_^−^ ions to diffuse to counter electrode and it hence would also have large *R*_D_. Therefore, *R*_D_ of samples C and D are smallest and *R*_D_ of samples A and F are largest, as sample A has the thickest TiO_2_ NP thin film and sample F has the highest density of TiO_2_ NPs in this study.

**Table 1:** **Characteristics of DSSCs using TiO**_**2 **_**nanoparticle thin film on the FTO glass fabricated by doctor blading method with and without compression treatment**

**Samples**	**Pressures (kg/cm**^**2**^**)**	***V***_**OC**_**(V)**	***J***_**SC**_**(mA/cm**^**2**^**)**	**F.F (%)**	**η (%)**	***R***_**Pt**_**(Ω)**	***R***_**K**_**(Ω)**	***R***_**D**_**(Ω)**
A	None	0.72	10.36	54.36	4.97	4.16	12.05	9.7
B	61	0.71	13.69	56.06	5.41	4.36	10.97	5.9
C	131	0.71	14.56	54.71	5.62	5.08	9.56	3.6
D	279	0.70	15.11	56.32	5.94	4.25	9.38	3.9
E	558	0.70	14.65	53.18	5.46	4.39	10.11	5.3
F	858	0.71	12.55	53.82	4.83	5.38	12.77	8.3

T.H. Meen and J.K. Tsai. *et.al.* Table 1.

**Figure 9:**
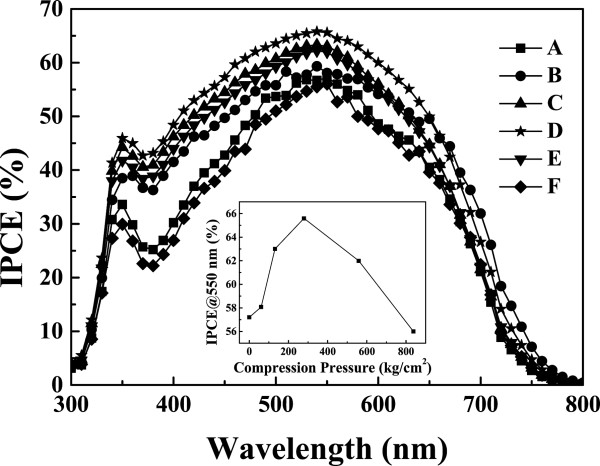
**IPCE characteristics of DSSCs using TiO**_**2 **_**nanoparticles photoanode method with and without compression treatment.** Curve A is the as-deposited film, and curves B to F are the after mechanical compression at various pressures, 61, 131, 279, 558, and 858 kg/cm^2^. Inset: the IPCE at wavelength of 550 nm as a function of the compression pressure.

Figure 9 shows the incident monochromatic photon-to-current conversion efficiency as a function of photon wavelength ranging from 300 to 800 nm. Sample D has the maximum IPCE among all the samples. In general, the photocurrent depends on the number of photo-excited electrons which is related with the absorption capacity of dye molecules, the recombination rate between electrons and oxidized dye molecules, and the redox series in the electrolyte. Increase of light traveling distance in photoanode thin film would improve efficiency of photo excitation; however, thick photoanode thin film would also enhance the recombination rate. Thus, to have high IPCE compromise among the increase of optical absorption, the reduction of recombination rate and the improvement of effective carrier transport are necessary. For example, sample A possesses the best absorption spectra among all the samples but its IPCE is not the highest one. The result indicates that the photo-excited electrons generated after the absorption of incident light by dye molecules cannot effectively transport to anode electrode due to high recombination rate and long passing distance (thick TiO_2_ NP thin film) that cause high *R*_K_ and R_*D*_ observed in Figure 8. The inset of Figure 9 shows the IPCE at wavelength of 550 nm as a function of the compression pressure. The IPCE of sample D is 8% larger than that of sample A.

**Figure 10:**
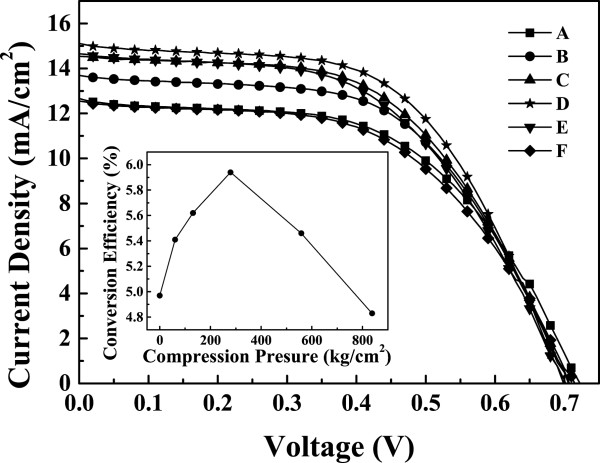
**Current densities against voltage (J-V) characteristics of DSSCs using TiO**_**2 **_**nanoparticles photoanode with and without compression treatment.** Curve A is the as-deposited film, and curves B to F are the after mechanical compression at various pressures, 61, 131, 279, 558, and 858 kg/cm^2^. Inset: the photoelectric conversion efficiency as a function of the compression pressure.

Figure 10 shows the results of the photocurrent-voltage characteristics of samples A to F under AM 1.5 G simulated sunlight. The photovoltaic properties of DSSCs are also summarized in Table 1. The open circuit voltage (*V*_OC_) and Fill factor of each sample is almost the same, indicating that the mechanical compression only changes the inter-particle distance. Sample D, whose TiO_2_ NP thin film was compressed with the pressure of 279 kg/cm^2^, has the highest short circuit current density (*J*_SC_) of 15.11 mA/cm^2^ and the photoelectric conversion efficiency (η) of 5.94%. In contrast, sample A, whose TiO_2_ NP thin film did not treat with any mechanical compression, has the highest absorbance but has the lowest short circuit current density (*J*_SC_) of 10.36 mA/cm^2^ and has only 4.97% photoelectric conversion efficiency (η). The result indicates that tuning inter-particle distance by compression of TiO_2_ NP thin films is an effective method to improve the performance of DSSCs. There exist an optimal inter-particle distance so that the IPCE and current density is maximized. Thus, obtaining optimal inter-particle distance of TiO_2_ nanoparticles is essential to fabricate high performance DSSCs.

## Conclusions

In this study, the compression of TiO_2_ NP thin film was tested to investigate the effect of film density. The results indicate that the performance of DSSC is compromised among the increase of optical absorption, the reduction of recombination rate, and the improvement of effective carrier transport. Therefore, the distance between TiO_2_ NPs is essential to tune the performance of DSSCs. The distance can be characterized by the porosity of TiO_2_ NPs films as analyzed by the ImageJ program. The results demonstrate that as the compression pressure increases the porosity decreases hence close-packing TiO_2_ NPs. The DSSC fabricated by the TiO_2_ NP thin film compressed at the pressure of 279 kg/cm^2^ has the smallest charge transport resistance at the TiO_2_/dye/electrolyte interface, the highest incident monochromatic photon-to-current conversion efficiency, the largest short circuit photocurrent density, and the highest photoelectric conversion efficiency among all the samples. Compared to the DSSC fabricated by the TiO_2_ NP thin film without any compression treatment, the conversion efficiency increases from 4.97% to 5.94% that is improved more than 19.5%. The compression treatment would not significantly change the *V*_OC_ indicating that the treatment only changes inter-particle distance. The results of the photocurrent-voltage characteristics, EIS, and IPCE measurement are consistent with each other. The compression treatment is an effective method to fabricate crack-free and high-quality TiO_2_ NP thin film for high performance DSSC.

## Abbreviations

DSSC: Dye-sensitized solar cells; EIS: Electrochemical impedance spectroscopy; FE-SEM: Field emission scanning electron microscopy; FTO: Fluorine-doped-tin oxide; IPCE: Incident photon-to-current conversion efficiency; ITO: Indium tin oxide; NPs: Nanoparticles; TiO_2_: Titanium dioxide; UV–vis: Ultraviolet–visible.

## Competing interests

The authors declare that they have no competing interests.

## Authors’ contributions

JKT designed the work and wrote the manuscript. YST carried out the preparation of samples, UV–vis absorption, and I-V measurements. WDH carried out the measurement and analysis of EIS. THM, TCW, and CSJ helped in carrying out the FE-SEM and IPCE measurements. All authors read and approved the final manuscript.
